# Regulating Te Vacancies through Dopant Balancing via Excess Ag Enables Rebounding Power Factor and High Thermoelectric Performance in p‐Type PbTe

**DOI:** 10.1002/advs.202100895

**Published:** 2021-08-13

**Authors:** Hanhwi Jang, Jong Ho Park, Ho Seong Lee, Byungki Ryu, Su‐Dong Park, Hyeon‐Ah Ju, Sang‐Hyeok Yang, Young‐Min Kim, Woo Hyun Nam, Heng Wang, James Male, Gerald Jeffrey Snyder, Minjoon Kim, Yeon Sik Jung, Min‐Wook Oh

**Affiliations:** ^1^ Department of Materials Science and Engineering Korea Advanced Institute of Science and Technology (KAIST) Daejeon 34141 Republic of Korea; ^2^ Electrical Materials Research Division Korea Electrotechnology Research Institute Changwon 51543 Republic of Korea; ^3^ School of Materials Science and Engineering Kyungpook National University Daegu 41566 Republic of Korea; ^4^ Department of Energy Science Sungkyunkwan University Suwon 16419 Republic of Korea; ^5^ Energy Efficiency Materials Center Korea Institute of Ceramic Engineering and Technology Jinju 52851 Republic of Korea; ^6^ Department of Mechanical, Materials, and Aerospace Engineering Illinois Institute of Technology Chicago IL 60616 USA; ^7^ Department of Materials Science and Engineering Northwestern University Evanston IL 60208 USA; ^8^ Department of Materials Science and Engineering Hanbat National University Daejeon 34158 Republic of Korea

**Keywords:** defect engineering, power factor, re‐dissolution, thermal conductivity, thermoelectrics

## Abstract

Thermoelectric properties are frequently manipulated by introducing point defects into a matrix. However, these properties often change in unfavorable directions owing to the spontaneous formation of vacancies at high temperatures. Although it is crucial to maintain high thermoelectric performance over a broad temperature range, the suppression of vacancies is challenging since their formation is thermodynamically preferred. In this study, using PbTe as a model system, it is demonstrated that a high thermoelectric dimensionless figure of merit, *zT* ≈ 2.1 at 723 K, can be achieved by suppressing the vacancy formation via dopant balancing. Hole‐killer Te vacancies are suppressed by Ag doping because of the increased electron chemical potential. As a result, the re‐dissolution of Na_2_Te above 623 K can significantly increase the hole concentration and suppress the drop in the power factor. Furthermore, point defect scattering in material systems significantly reduces lattice thermal conductivity. The synergy between defect and carrier engineering offers a pathway for achieving a high thermoelectric performance by alleviating the power factor drop and can be utilized to enhance thermoelectric properties of thermoelectric materials.

## Graphical abstract

Charge compensation by intrinsic defects poses a big challenge for active tuning of the carrier concentration via temperature‐activated doping. By regulating Te vacancies in a p‐type PbTe via Ag doping, it is demonstrated that a synergistic interplay between a rebounding power factor and suppressed lattice thermal conductivity can improve thermoelectric performance.

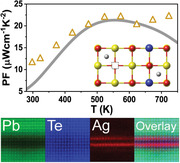

## 1 Introduction

A direct conversion of heat to electrical energy using thermoelectric (TE) devices has been considered as an efficient energy harvesting technique to address current shortage of worldwide energy.^[^ ^1^ ^]^ The conversion efficiency of TE materials depends on the dimensionless figure of merit (*zT*), defined as $zT=\frac{S^{2}\sigma }{\kappa }T$, where *T*, *S*, *σ*, and *κ* are the absolute temperature, Seebeck coefficient, electrical conductivity, and thermal conductivity of the TE material, respectively.^[^ ^1^, ^2^ ^]^ From this equation, it is important to increase both *S* and *σ* while decreasing *κ* in order to develop highly efficient TE materials.

Several TE materials including Bi_2_Te_3_,^[^ ^3^ ^]^ Zintl phases,^[^ ^4^ ^]^ GeTe,^[^ ^5^ ^]^ SnSe,^[^ ^6^ ^]^ AgSbTe_2_,^[^ ^7^ ^]^ Mg_2_Si,^[^ ^8^ ^]^ and PbTe^[^ ^9^ ^]^ are known to exhibit high TE performance. In particular, PbTe‐based TE materials have been extensively studied owing to their intrinsically low *κ* and high *σ*.^[^ ^10^ ^]^ Strategies, such as distorting the density of states,^[^ ^11^ ^]^ inducing band convergence,^[^ ^9c^ ^]^ designing a hierarchical structure,^[^ ^9b^ ^]^ constructing a strained lattice,^[^ ^12^ ^]^ and optimizing the carrier concentration,^[^ ^13^ ^]^ have been proposed to increase the TE performance of PbTe.

Typically, introducing point defects, such as substitutional or interstitial atoms, to the matrix is a common method to manipulate the properties of TE materials.^[^ ^14^ ^]^ However, this strategy is frequently hindered by intrinsic vacancies, which are often detrimental to the TE properties and are difficult to control.^[^ ^15^ ^]^ Few studies have been conducted to improve the TE properties by controlling the vacancy concentration near room temperature.^[^ ^16^ ^]^ However, it is challenging to regulate vacancy formation at high temperatures, as it is thermodynamically stable.^[^ ^14^, ^17^ ^]^ Therefore, a novel design rule is required to suppress the spontaneous formation of vacancies.

Ag has been considered a PbTe dopant candidate owing to the high TE performance of lead–antimony–silver telluride compounds.^[^ ^18^ ^]^ Furthermore, it has limited solubility in PbTe; thus, it can form precipitates in dislocations or grain boundaries in the PbTe matrix to suppress phonon transport.^[^ ^19^ ^]^ It has been reported that Ag defects in PbTe can act as donor‐like interstitials under certain conditions.^[^ ^20^ ^]^ Donor‐like interstitial Ag atoms can increase the electron chemical potential and formation energy of donor defects, such as *V*_Te_^2+^. From the above results, we hypothesized that the temperature‐dependent solubility of Ag would prevent the formation of n‐type vacancies at high temperatures.

As a proof‐of‐concept, Na‐doped PbTe was used as a model system with significant Te vacancy formation and degraded TE properties. Thereafter, we intentionally doped excess Ag as an interstitial donor into the Na‐doped PbTe. Because the Ag dopant triggers the temperature‐activated doping of Na by regulating the formation of Te vacancies, we can actively manipulate the carrier concentration of the Na_0.04_Pb_0.96_Ag*_y_* Te (0 ≤ *y* ≤ 0.05) systems at high temperatures. Although the simultaneous doping of acceptors and donors may seem ineffective, we demonstrate that it is a promising strategy to effectively decrease *κ* while alleviating the power factor (PF) drop at high temperatures. The temperature‐activated doping caused by the re‐dissolution of Na above 623 K supplies excess holes to PbTe, resulting in temperature‐independent plateaus in both *σ* and *S*. Both annihilation of the formation of *V*_Te_ and temperature‐activated doping yield a rebounding PF; thus, the electronic transport properties of PbTe are preserved in the high‐temperature range.

As for the thermal properties, the counteracting Ag dopant further reduces the electronic *κ* (*κ*_e_) by donating electrons to the PbTe matrix and decreasing the hole concentration at room temperature. The additional incorporation of Na and Ag at high temperatures effectively scatters heat‐carrying phonons, which significantly reduces the lattice *κ* (*κ*_latt_) to as low as 0.44 Wm^−1^ K^−1^ at 723 K. The rebounding PF combined with the suppressed *κ* significantly increased *zT*, reaching *zT*_max_ of 2.1, at 723 K in Na_0.04_Pb_0.96_Ag_0.02_Te. This is 31.3% higher than that of Na_0.04_Pb_0.96_Te owing to the concurrent enhancement of the electronic and thermal transport properties. The temperature‐averaged *zT* (*zT*_avg_) of quaternary PbTe exceeded that of ternary PbTe by 11.2%, which is comparable to that of state‐of‐the‐art high‐performance PbTe materials at operational temperatures.

## 2 Results and Discussions

### 2.1 Enhancing TE Performance by Ag Addition

**Figure 1:**
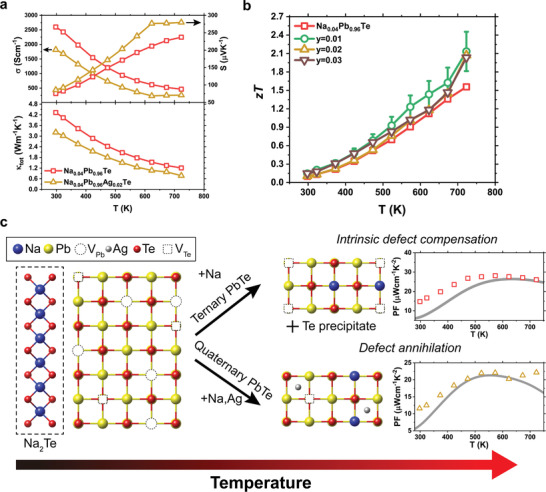
Rebounding power factor observed in the quaternary PbTe. a) Temperature‐dependent electrical conductivity (*σ*), Seebeck coefficient (*S*), and total thermal conductivity (*κ*_tot_) of Na_0.04_Pb_0.96_Te and Na_0.04_Pb_0.96_Ag_0.02_Te. b) *zT* of Na_0.04_Pb_0.96_Te and Na_0.04_Pb_0.96_Ag_y_Te as a function of temperature. The uncertainty of *zT* measurement of 15% is shown with error bars. c) Schematic diagram showing a different defect formation when the Na re‐dissolution occurs at high temperatures. The ternary PbTe compensates incorporated Na atoms by forming tellurium vacancies (*V*_Te_), and Te metal precipitates out of the matrix. However, the charge compensation by intrinsic defects is suppressed in the quaternary PbTe. The measured PF values of the quaternary PbTe deviate from the theoretical expectation starting from 623 K, showing the rebounding PF characteristics.

Our preliminary experiment to determine the optimum Na concentration in Na*_x_* Pb_1‐_ *_x_* Te (Figure S1, Supporting Information) achieved the ideal TE properties at *x* = 0.04, with a *zT*_max_ of 1.6, at 723 K, which is consistent with previous results.^[^ ^21^ ^]^ Therefore, *x* = 0.04, was selected as the starting composition for further study. From the X‐ray diffraction (XRD) analysis, all samples of PbTe doped with Na and Ag were identified as crystallizing in the NaCl‐type crystal structure, and no secondary phase was detected within the detection limit (Figure S2, Supporting Information). Small amounts of Na_2_Te and Ag_2_Te precipitates were observed in the quaternary system (see Figures S3 and S4, Supporting Information). The temperature‐dependent TE properties of Na_0.04_Pb_0.96_Te and Na_0.04_Pb_0.96_Ag_0.02_Te are shown in **Figure** **1**a. The 2% Na‐doped PbTe exhibited *σ* ≈ 2600 S cm^−1^ at room temperature, which monotonically decreased to 460 S cm^−1^ at 723 K; this is typical of a highly degenerate semiconductor. The above characteristic behavior of Na_0.04_Pb_0.96_Te was also reflected by the temperature dependence of *S* (≈80 µV K^−1^ at room temperature and 240 µV K^−1^ at 723 K), which was linearly proportional to the temperature.

The *σ* trend of 2% Na‐doped PbTe with an additional 1% Ag was similar to that without Ag, showing lower values than the ternary system of *σ* ≈ 1800 S cm^−1^ at room temperature and 260 S cm^−1^ at 723 K. However, *S* increased from 85 µV K^−1^ at room temperature to 280 µV K^−1^ at 723 K. The decrease in *σ* and increase in *S*, caused by Ag doping, is owing to the reduced hole concentrations due to the counter‐doping effect of Ag with Na, where interstitial Ag (Ag_i_) acts as a hole‐killer defect, and the donor‐like Ag_2_Te precipitates also form. This is consistent with the calculation results reported by Ryu et al., which predicted that Ag_i_ is the most preferable n‐type point defect in p‐type PbTe grown under Te‐rich conditions.^[^ ^20^ ^]^ The solubility of Ag was less than 1 at%;^[^ ^22^ ^]^ thus, the Ag_2_Te precipitates preferably form in the quaternary. As mentioned in the previous paragraph, Ag_2_Te precipitates were observed. These precipitates also caused to the formation of donor‐like *V*_Te_ and reduced the hole concentration. The formation of Ag_i_ and Ag_2_Te is also supported by carrier concentration analyses, which will be addressed in a later section. Temperature‐insensitive behaviors of *S* and *σ* were observed above 623 K for Na_0.04_Ag_0.02_Pb_0.96_Te, with 1% variation in *S* and 10% variation in *σ* upon a 100 K increase.

Conversely, a significant decrease in *κ*_tot_ upon Ag doping was observed across a wide temperature range, as evidenced by the 40% decrease from 1.21 to 0.77 Wm^−1^ K^−1^ at 723 K for the ternary and quaternary systems, respectively. This overall decrease can be attributed to the suppressed electronic and lattice *κ* in the quaternary system compared to that of the ternary system. The *zT* values in Figure 1b show that *zT* increased owing to the temperature‐insensitive behavior of *S* and *σ* in Na_0.04_Pb_0.96_Ag_y_Te; thus, *zT* above 623 K increased abruptly to a *zT*_max_ of ≈2.1, at 723 K. Figure 1c is a schematic diagram showing the origin of the temperature‐insensitive transport properties and high TE performance. The Na_2_Te precipitate dissolves into the PbTe matrix at high temperatures for both the ternary and quaternary PbTe, which was confirmed in various experimental investigations shown later in this study. However, in the ternary system, the incorporated Na atoms at the Pb sites could not provide extra holes because electrons from intrinsic defects (*V*_Te_) compensated the holes from Na_Pb_. This is evidenced by the formation of metallic Te precipitates at high temperatures in the ternary system.^[^ ^17^ ^]^ This intrinsic defect (*V*_Te_) creation is suppressed in the quaternary PbTe, allowing an abrupt increase of the hole concentration. Owing to *V*_Te_ defect annihilation by Ag, the decrease in PF is prevented at high temperatures; thus, improving *zT* values.

The achieved peak *zT* at 723 K was comparable to that of state‐of‐the‐art p‐type TE materials (Figure S5, Supporting Information), and it was 31.3% higher than that of Na_0.04_Pb_0.96_Te. *zT*_avg_ across the investigated temperature range was also estimated and compared with those of other p‐type PbTe‐based materials (Figure S5 and Table S1, Supporting Information). Because the *zT* of PbTe below 473 K is lower than that of conventional Bi_2_Te_3_‐based compounds,^[^ ^2^, ^23^ ^]^ Bi_2_Te_3_‐based compounds are used in TE modules where the conditions involve a cold‐side temperature of 300 K and hot‐side temperature of 473 K.^[^ ^24^ ^]^ Thus, within the practical temperature range of 473–723 K, *zT*_avg_ of our sample is competitive with those of state‐of‐the‐art p‐type TE materials.

### 2.2 Behavior of Incorporated Dopants in PbTe

**Figure 2:**
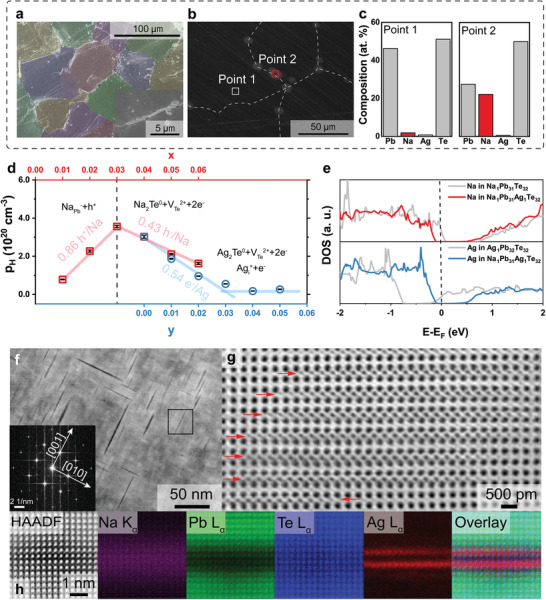
Characterization of Na_0.04_Pb_0.96_Ag_y_Te. a) False color low‐magnification SEM image of the fracture surface of Na_0.04_Pb_0.96_Ag_0.02_Te_._ The average grain size is ≈80 µm. Inset is the high‐magnification SEM image of the grain boundary in (a), showing the segregation of expelled Na at the grain boundary. b) Typical SEM image of the polished surface of Na_0.04_Pb_0.96_Ag_0.02_Te. White dots (Na‐rich phases) exist at grain boundaries. c) EDX analysis results of the matrix and precipitates at the grain boundary. d) Measured Hall carrier concentration of Na*_x_* Pb_1−_ *_x_* Te (*x* = 0.01–0.06) and Na_0.04_Pb_0.96_Ag*_y_* Te (*y* = 0–0.05). e) Partial densities of states of Na and Ag in ternary and quaternary PbTe. f) Low‐magnification HAADF STEM image of the Na_0.04_Pb_0.96_Ag_0.02_Te in the [100] zone axis. Inset is the fast Fourier transform of the boxed region. Needle‐like nanostructures are Ag_2_Te precipitates and interstitial Ag atoms. g) Atom‐resolved ABF STEM image of interstitial Ag atoms in the PbTe matrix. Red arrows indicate interstitial Ag atoms. h) Atom‐resolved HAADF STEM image and corresponding atomic‐resolution EDX mapping of Na, Pb, Te, and Ag. Na signals were excluded from the overlay image for clarity.

The scanning electron microscope (SEM) images of *y* = 0.02 sample is shown in **Figure** **2**a,b. From the SEM images, clear segregation of precipitates can be observed near the grain boundaries. This precipitation was identified as Na‐rich regions, meaning that the solid solubility of Na in PbTe is below 2 at% when Ag is simultaneously doped (Figure 2c). The observed average grain size was ≈80 µm, which is relatively large compared with that of spark‐plasma‐sintered materials because of the long hot‐pressing process at a high temperature.^[^ ^9^, ^25^ ^]^ Thus, the effect of grain boundary scattering on reducing the lattice *κ* (*κ*_latt_) is expected to be negligible. The expelled Na formed Na_2_Te at the grain boundaries.

We explore the doping characteristics of Na and Ag in PbTe. The hole concentration increased with Na content in the ternary Na‐doped PbTe up to *x* = 0.03, because Na_Pb_ defects act as acceptors.^[^ ^26^ ^]^ However, heavier Na doping over *x* = 0.03 decreased the hole concentration (Figure 2d). The solubility of Na in PbTe is ≈1.4 at%.^[^ ^27^ ^]^ Excess Na doping over the solubility limit of Na in PbTe causes to the formation of the Na_2_Te second phase, as well as *V*_Te_ donor defects, resulting in a decrease in the hole concentration. The Ag co‐doping in PbTe decreases the hole concentration owing to hole‐killer Ag_i_ defects and donor‐like Ag_2_Te. The doping efficiency of Ag is estimated to be 0.54 e^−^/atom, which is larger than that of excess Na doping (0.43 e^−^/atom), even though *V*_Te_ originates from Ag_2_Te and Na_2_Te in the quaternary and ternary systems, respectively. This discrepancy may result from electron donation from both Ag_i_ and *V*_Te_ formed by Ag_2_Te.

We conducted density functional theory (DFT) calculations to understand the doping characteristics of Na and Ag. The partial densities of states of Na and Ag in the ternary and quaternary systems are shown in Figure 2e. The p‐type and n‐type doping characteristics, as indicated by the position of the Fermi level, were confirmed for Na_Pb_ and Ag_i_ in the ternary system, respectively. However, the Fermi level is located at the valence band maximum (VBM) for quaternary PbTe. The simultaneous doping of Na_Pb_ and Ag_i_ defects cannot provide carriers if the concentration and ionization of both dopants are the same. The counter‐doping effects of Na_Pb_ and Ag_i_ were confirmed by DFT calculations.

In addition, we confirmed the Na_Pb_ and Ag_i_ defects in the quaternary PbTe by aberration‐corrected scanning transmission electron microscopy (STEM) techniques. Figure 2f shows a low‐magnification high‐angle annular dark‐field (HAADF) STEM image of Na_0.04_Pb_0.96_Ag_0.02_Te viewed along the [100] zone axis. HAADF is sensitive to the atomic number (*Z*) contrast, producing a brighter image for heavier elements.^[^ ^28^ ^]^ Here, needle‐like precipitates appear darker than the matrix in the HAADF image, implying that the constituent elements have a lower atomic mass than those of the matrix. Moreover, we found that these precipitates preferentially grow in directions parallel to <100>, which is a unique characteristic of Ag‐related nanostructures in PbTe.^[^ ^13^, ^29^ ^]^ These nanostructures were uniformly distributed throughout the sample (Figure S6, Supporting Information). The atom‐resolved annular bright‐field (ABF) STEM image in Figure 2g shows that these needles consist of several interstitial atoms. The morphology of these ordered nanoclusters is similar to that of Cu interstitials in PbCu_0.0075_Se.^[^ ^30^ ^]^ The atomic‐resolution energy‐dispersive X‐ray spectroscopy (EDX) mapping in Figure 2h corroborates that Ag atoms are located at interstitial sites, while Na atoms substitute Pb sites, which is consistent with the Hall measurement results.

**Figure 3:**
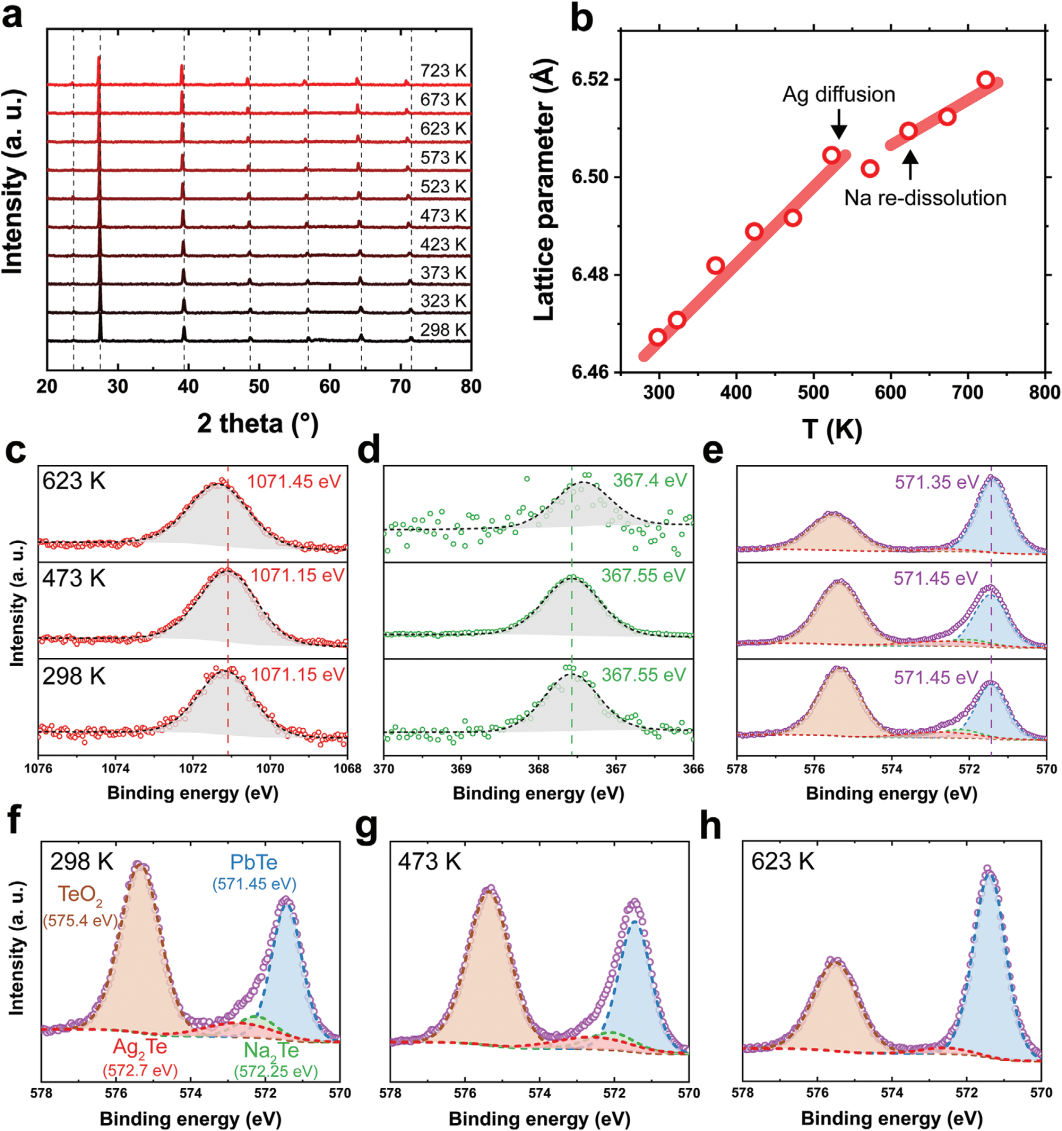
Observation of the re‐dissolution via in situ characterizations. a) High‐temperature XRD patterns of Na_0.04_Pb_0.96_Ag_0.02_Te and b) calculated lattice parameters from (a). Solid red lines are guide to the eye. The temperatures for Na re‐dissolution and Ag redistribution from DSC are shown by arrows. c–e) In situ XPS spectra of the c) Na 1s orbital, d) Ag 3d orbital, and e) Te 3d orbital. Peak shifts observed in (c) imply a change of binding energy of Na. f–h) Deconvoluted XPS spectra of the Te 3d orbital measured at f) 298, g) 473, and h) 623 K. The reduced peak intensities of Na_2_Te and Ag_2_Te at 623 K are owing to the increased solubility of Na and Ag in the PbTe matrix at high temperatures.

The extrinsic carrier generation characteristics of Na_0.04_Pb_0.96_Ag_0.02_Te were further studied by high‐temperature Hall measurements (Figures S7 and S8, Supporting Information). The activation energy of the excess carriers (Δ*E*_ac_) was determined in the ascending region.^[^ ^30^ ^]^ The value is 0.37 eV, which is larger than 0.17 eV that observed for Na‐doped PbTe.^[^ ^9c^ ^]^ The larger value is owing to the redistribution of Ag at elevated temperatures. The re‐dissolution of Na consists of five processes: Na_2_Te dissociation, Na diffusion, Na_Pb_ substitution, *V*_Te_ formation, and hole generation. However, the redistribution of Ag requires four extra steps: Ag_2_Te dissociation, Ag diffusion, interstitial site occupation, and electron generation. These additional carrier generation processes increase the activation energy. Both redistribution and re‐dissolution are supported by in situ X‐ray photoelectron spectroscopy (XPS) (**Figure** **3**) and differential scanning calorimetry (DSC) characterizations (Figure S9, Supporting Information).

Thereafter, we discuss that *V*_Te_ formation can be suppressed in the quaternary system. The re‐dissolution process at high temperatures can be described by the following defect equation:^[^ ^17^ ^]^

(1) $$Na_{y}+Pb_{1-x}\mathrm{Te}\to Na_{y}Pb_{1-x}Te_{1-\delta }+\delta \mathrm{Te}$$

The activation is accompanied by the formation of *V*_Te_ defects and Te precipitates induced by the re‐dissolution of Na at elevated temperatures. However, the quaternary system increases the chemical potential of electrons compared to that of the ternary system owing to n‐type Ag_i_ defects. The formation energy of *V*_Te_ increased in this circumstance.^[^ ^20^ ^]^ As a result of the suppressed *V*_Te_ formation, metallic Te precipitates were not observed in high‐temperature XRD and XPS (Figure 3). The Δ*E*_ac_ of Ag‐doped PbTe is 0.21 eV.^[^ ^26a^ ^]^ This implies that the mechanism for the re‐dissolution of the quaternary system is different from that of ternary PbTe.

A supply of charge carriers from extrinsic repositories has also been reported in Cu‐doped PbSe,^[^ ^30^ ^]^ Ag‐doped PbTe,^[^ ^13^ ^]^ and Cu‐doped PbTe,^[^ ^31^ ^]^ which are related to the increase in the solid solubility with temperature. Similarly, with increasing temperature, Ag can be easily distributed throughout the PbTe lattice.^[^ ^13^, ^20^ ^]^ Calorimetric analysis also supports this redistribution and re‐dissolution. Two exothermic peaks occurred for the quaternary PbTe starting near 500 and 600 K, which were preserved after the first measurement (Figure S9, Supporting Information). The initiation temperature range for the decomposition of Ag_2_Te and redistribution of Ag has been estimated to be 450–550 K,^[^ ^13^, ^20^ ^]^ while the dissolution of excess Na at grain boundaries was shown to begin at 620 K in Na‐ and Sr‐doped PbTe.^[^ ^9a^ ^]^ Additionally, the plateau in the electrical properties began at 623 K for highly Na‐doped PbTe (Figure S1, Supporting Information). Therefore, the first and second exothermic peaks at 500 and 600 K correspond to the redistribution of Ag and dissolution of Na, respectively.

The relationship between S and the hole concentration was obtained from electronic structure calculations using DFT and the Boltzmann transport equation (Figure S10, Supporting Information). The theoretical values are in good agreement with those of previous reports.^[^ ^11^, ^32^ ^]^ The data obtained in this work (Na‐doped as well as Na and Ag co‐doped PbTe) also fall on the same line, illustrating that Na and Ag doping does not significantly change the electronic structure characteristics of PbTe.^[^ ^30^ ^]^

### 2.3 TE Properties of Na_0.04_Pb_0.96_Ag*_y_* Te

**Figure 4:**
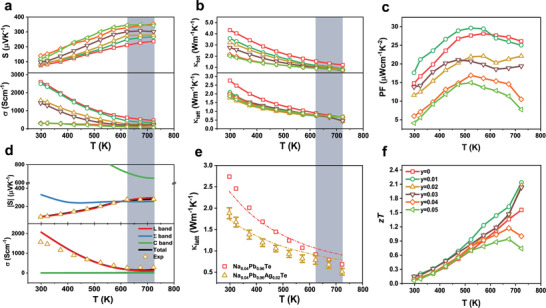
Temperature‐dependent thermoelectric properties of Na_0.04_Pb_0.96_Ag_y_Te. a) Seebeck coefficient (*S*) and electrical conductivity (*σ*), b) total thermal conductivity (*κ*_tot_, solid lines), and lattice thermal conductivity (*κ*_latt_, broken lines). c) Power factor (PF), and f) dimensionless figure of merit (*zT*). d) Theoretical Seebeck coefficient and electrical conductivity obtained by solving the Boltzmann transport equation using the three‐band model. e) Experimental lattice thermal conductivity and prediction from the Debye–Callaway model of the ternary and quaternary PbTe. The error bar denotes the standard deviation from five measurements. The deviations stem from Ag redistribution and Na re‐dissolution at elevated temperatures. f) The same symbols indicate the corresponding concentrations of Ag. Blue shades indicate the region where a plateau appears.

The effect of Ag concentration on the temperature‐dependent TE properties of Na_0.04_Pb_0.96_Ag_y_Te (0 ≤ *y* ≤ 0.05) was subsequently studied. With increasing Ag concentration, *σ* decreased, whereas *S* increased, as shown in **Figure** **4**a. In the case of *y* = 0.04 and 0.05, the decrease in *σ*, especially at low temperatures, was significantly larger than the increase in *S*, causing a lower PF than those of the samples with low Ag concentrations. A plateau in the electrical transport properties at 623 K was observed for the three lower compositions (*y* = 0.01, 0.02, and 0.03). The contribution of the heavy band to the electrical transport is limited in the larger Ag compositions, resulting in a plateau only in the lower compositions. A detailed analysis is discussed in the next section.

The temperature dependence of the total thermal conductivity (*κ*_tot_) and *κ*_latt_ of Na_0.04_Pb_0.96_Ag_y_Te is plotted in Figure 4b. *κ*_latt_ was determined by subtracting *κ*_e_ estimated by the Wiedemann–Franz equation, in which the Lorenz number was calculated using the single parabolic band assumption.^[^ ^33^ ^]^ *κ*_tot_ is attributed to both *κ*_e_ and *κ*_latt_ for the ternary and the lower compositions over the entire temperature range, while both *κ*_e_ and *κ*_latt_ contributed to *κ*_tot_ at high temperatures for the larger compositions. *κ*_tot_ decreased with increasing Ag concentration because of the decrease in hole concentration induced by the counter‐doping effect of Ag. Additionally, the reduction of *κ*_latt_ at elevated temperatures with Ag co‐doping is owing to the increased point defect scattering caused by the redistribution of Ag and Na at elevated temperature. The temperature‐dependent *κ*_latt_ behavior was similar for all Ag‐doped samples, except for a further decrease at high temperatures for *y* = 0.01–0.03. This main difference in *κ*_latt_ results from the *σ* plateau at high temperatures, which did not occur for *y* = 0.04 and 0.05. This difference might also arise from the inaccurate estimation of the Lorenz number for *y* = 0.04 and 0.05, as these samples may experience bipolar conduction at high temperatures because of the low hole concentration. Thus, excess Ag‐doped samples (*y* = 0.1 and 0.15) exhibit non‐degenerate characteristics in electronic properties and p‐ to n‐type transition near 400 K owing to severe bipolar conduction (Figure S11, Supporting Information). However, there is a low chance for bipolar conduction for the lower compositions because of the high hole concentration (on the order of 10^20^ cm^−3^) above 600 K.^[^ ^30^ ^]^

The changes in PF and *zT* with different Ag concentrations at different temperatures are shown in Figure 4c,f. The counter‐doping effect of Ag was also reflected by the PF, as the temperature corresponding to PF_max_ decreased for higher Ag contents. Furthermore, PF was nearly preserved and rebounded for the lower compositions (*y* = 0.01–0.03) above the first maxima owing to the electronic transport plateau. Combining the decreased *κ*_tot_ and rebounded PF, Na_0.04_Pb_0.96_Ag_y_Te exhibits a high TE performance, with *zT*_max_ exceeding 2.0 for *y* = 0.01–0.03 at 723 K. These high *zT* values originate from the increased hole concentration owing to the re‐dissolution of Na and suppressed *V*_Te_ formation induced by the redistribution of Ag at high temperatures.

Systematic calculations using the Boltzmann transport equation were performed to understand how Na re‐dissolution results in plateaus in the electronic transport properties. The detailed calculation procedures are provided in the Supporting Information. Figure 4d shows the contributions of the light hole (L), heavy hole (Σ), and conduction (C) bands to *S* and *σ*. The contribution of the L band to *S* is highly sensitive to temperature. Hence, *S* increased with temperature when considering only the L band contribution, which is not consistent with the quasi‐static *S* observed above 600 K. However, this behavior can be understood by considering that the contribution of the temperature‐insensitive Σ band dominates the total *S* in the given temperature range, thus suppressing the increase in *S* at high temperatures. The change in the property‐dominant band near 600 K is also reflected by *σ*. Similarly, the largest contribution to *σ* below 600 K is from the L band, which rapidly decreases with increasing temperature. The temperature dependence of *σ* from the Σ band increases with temperature, which compensates for the decrease in *σ* from the L band above 600 K.

Furthermore, it was also found that temperature‐activated doping is critical for band convergence, causing the Σ band to contribute to the electronic transport properties and preserving PF at elevated temperatures. Because the relative positions of the L and Σ bands are changed and the TE transport properties are affected by band convergence starting from 600 K, it proves that temperature‐activated doping occurs in the quaternary PbTe system. Without the re‐dissolution of Na, the quaternary system had a significantly lower hole concentration than that of the ternary system. Thus, the Fermi level of the quaternary system would be located closer to the edge of the L band. If this were the case, the temperature at which the L and Σ bands were converged would be higher than 600 K, which has been widely reported for highly degenerate PbTe systems.^[^ ^9^, ^34^ ^]^ However, the increased hole concentration due to the re‐dissolution of Na at elevated temperatures drags the Fermi level to much lower energy than valence band maximum (VBM) of the band at the L point, which facilitates band convergence at lower temperatures than without Na re‐dissolution.

We found that the re‐dissolution process can further decrease *κ*_latt_ in the quaternary system. The contributions of various scattering mechanisms on *κ*_latt_ were calculated using a modified Debye–Callaway model and are plotted together in Figure 4e. Without boundary scattering, the Umklapp process with a dislocation core, dislocation strain, and point defect scattering describe the behavior of *κ*_latt_ below 500 K with a satisfactory fitting. The high density of dislocations in the samples is shown in Figures S12 and S13, Supporting Information. However, *κ*_latt_ deviates from the theoretical prediction above ≈500 K, which may result from re‐dissolution and redistribution at elevated temperatures. The effect of intensified point defect (PD) scattering, originating from both Na_Pb_ and Ag_i_ formation at elevated temperature, explains the decrease in *κ*_latt_, A decrease in *κ*_latt_ owing to Na dissolution was also observed in Na‐ and Sr‐doped PbTe.^[^ ^9b^ ^]^ Moreover, the suppressed drop in *σ* increases *κ*_e_, which also reduces the contribution of *κ*_latt_ to *κ*_tot_ at high temperatures, given that *κ*_e_ accounts for 26.6% of *κ*_tot_ at 723 K.

### 2.4 In Situ Characterization of Na_0.04_Pb_0.96_Ag_0.02_Te

Here, we show that quaternary PbTe readily accepts Na_Pb_ without forming *V*_Te_ at high temperatures. Figure 3a shows the temperature‐dependent powder XRD patterns from 298 to 723 K. All peaks are indexed to the cubic PbTe at all examined temperatures, unlike the ternary PbTe that forms Te precipitates.^[^ ^17^ ^]^ *V*_Te_ is commonly formed to compensate for Na_Pb_ defects to prevent PbTe from increasing the hole concentration.^[^ ^17^ ^]^ As a result of vacancy formation, Te atoms are expelled from the matrix to form metallic Te precipitates at high temperatures.^[^ ^17^ ^]^ However, metallic Te was not found in the quaternary PbTe within the detection limit of XRD, whereas metallic Te was in the XRD pattern of the ternary PbTe.^[^ ^17^ ^]^ The lattice parameters calculated from in situ XRD also show evidence of Na incorporation at 623 K after re‐dissolution (Figure 3b). Typically, lattice parameters increase owing to thermal expansion with a certain slope. However, the slope decreases at 623 K because the ionic radius of Na^+^ (0.102 nm) is smaller than that of Pb^2+^ (0.119 nm).^[^ ^35^ ^]^ This temperature is consistent with the calorimetric analysis results where Ag redistribution and Na re‐dissolution occur at 500 and 600 K, respectively. From the in situ XRD, we also confirmed that the re‐dissolution of Na and the re‐distribution of Ag at elevated temperatures, as well as suppression of the *V*_Te_ and resultant metallic Te formation.

To examine the change in the binding states at high temperatures in the quaternary PbTe, in situ XPS measurements were conducted. Figure 3c shows a high‐resolution scan of the Na 1s orbital at 298, 473, and 623 K. The binding energies of Na were higher than those of metallic Na. Moreover, it was found that the peak position of Na shifted to a higher binding energy at 623 K compared to that at room temperature, owing to changes in the local chemical environment. An increase in binding energy occurs when chemical species lose electrons to their surroundings. This is reasonable because Te atoms are expected to attract Na atom electrons more easily when Na is octahedrally bonded with Te in the form of Na_Pb_‐Te than when Na is tetrahedrally bonded with Te in Na_2_Te. A similar observation was reported for Na*_x_* CoO_2_, where Na occupying different crystallographic sites showed a shift in the peak binding energy.^[^ ^36^ ^]^ In contrast to Na, lower binding energies of Ag compared to metallic Ag were observed. (Figure 3d). This discrepancy may arise from the differences in the bonding states of Na and Ag, where Na_Pb_ is bonded with Te, and Ag_i_ is intercalated to interstitial sites. As Na_Pb_^−^ is an acceptor and Ag_i_^+^ is a donor defect, we can expect an opposite behavior in the shift of the binding energy owing to discrepancy in the charge screening. Similar to Ag, the peak positions of the Te 3d orbital emerge at higher binding energies compared to that of metallic Te (Figure 3e) as Te attracts electrons from Pb. The re‐dissolution behavior was observed in the deconvoluted XPS spectra of the Te 3d orbital (Figure 3f–h). The binding energies of 571.45, 572.25, 572.7, and 575.4 eV are indexed to Pb—Te, Na—Te, Ag—Te, and Te—O bonds, respectively.^[^ ^37^ ^]^ At room temperature, Pb—Te, Ag—Te, and Na—Te bonds were observed until 473 K. However, the peak intensities of the Na—Te and Ag—Te bonds significantly decreased at 623 K, implying that these secondary phases dissolved into the PbTe matrix at high temperatures. The dissolution of Na‐ and Ag‐rich phases can provide Te atoms to PbTe, preventing the formation of *V*_Te_ (see Supporting Information Notes for detailed defect chemistry calculations). Consequently, evidence from transport property measurements, in situ characterization, and calorimetric analysis corroborate the re‐dissolution behavior of Na and redistribution of Ag in the quaternary PbTe.

## 3 Conclusions

In this study, we showed that both *S* and *σ* can exhibit plateaus when the re‐dissolution of Na occurred above 600 K. Additionally, the increased PD density significantly decreased *κ*_latt_. Our in situ characterization and calorimetric analysis showed that Na‐induced *V*_Te_ formation was suppressed by the redistribution of Ag defects at elevated temperatures. Moreover, the incorporation of Na increased hole concentration, causing the rebounding of *S* and *σ*. Theoretical estimation of the electronic transport properties revealed that the contribution of the heavy Σ band causes rebounding transport properties at elevated temperatures. As the Fermi level shifts to deep states in the valence band with temperature‐activated doping, the quaternary system undergoes band convergence such that heavy holes contribute significantly to the electronic transport properties. Hence, a *zT*_max_ value of ≈2.1, at 723 K was achieved. The temperature‐activated doping can be synergistically applied for band engineering to actively modulate the carrier concentration of materials without modifying their electronic structure; thus, developing high‐performance TE materials. Defect engineering, in which intrinsic defect formation is annihilated by extrinsic defect formation, such as Ag_i_, is critical for promoting temperature‐activated doping. Therefore, constructing an all‐scale hierarchical architecture to scatter mid‐wavelength phonons can also be implemented to further enhance the *zT* values at mid‐temperature ranges to improve *zT*_avg_.

## 4 Experimental Section

### Materials

Pure elemental Na cubes (99.9%), Pb shots (99.999%), Ag granules (99.99%), and Te shots (99.999%) were purchased from Alfa Aesar. All elements were used as received without further purification.

### Synthesis

For the synthesis of Na*_x_* Pb_1‐_ *_x_* Te and Na_0.04_Pb_0.96_Ag*_y_* Te, a conventional melting and quenching process was used. Stoichiometric amounts of Na, Pb, Ag, and Te were weighed and placed in a carbon‐coated quartz tube. Then, the tube was sealed using flame after achieving a base pressure of 10^−4^ Torr. A rocking furnace was used to ensure the chemical homogeneity of the material. Melting was performed at 1273 K for 10 h, and the samples were quenched with air. Further annealing at 900 K for 72 h was conducted to stabilize and homogenize the materials. The synthesized ingots were pulverized with an agate mortar and sintered with a hot press at 873 K and a uniaxial pressure of 200 MPa for 1 h.

### Measurement of TE Properties

*σ* and *S* values were simultaneously measured using a commercial apparatus (ZEM‐3, ULVAC). *κ* was calculated as follows: *κ* = *ρDC*_p_, where *ρ* is the density, *D* is the thermal diffusivity, and *C*_p_ is the specific heat capacity. We measured the TE properties only up to 723 K, which is comparable to other Na‐doped PbTe^[^ ^26^ ^]^ measurements, to prevent sublimation or evaporation of the samples. *ρ* was measured geometrically by dividing the total mass by the volume, and *D* was measured using a laser flash method (LFA‐457, Netzsch). A constant *C*_p_ value of 0.171 J g^−1^ K^−1^ was used for the calculations, which was experimentally measured by DSC (DSC200 F3, Netzsch) of binary PbTe at 723 K (Figure S14, Supporting Information). The thermoelectric properties were verified through independent parties, including Thermoelectric Total Solution at Sungkyunkwan University (SKKU) and Northwestern University (NWU) with the ZEM‐3 equipment for the electrical properties, and KAIST Analysis center for Research Advancement (KARA) and NWU with the LFA‐457 equipment for thermal properties (Figure S15, Supporting Information). We calculated *z*_int_ *T*_avg_ as an average *zT* value according to Kim et al.^[^ ^38^ ^]^ *z*_int_ and *T*_avg_ were calculated as $z_{\mathrm{int}}=\frac{\int_{T_{\mathrm{cold}}}^{723}z(T)dT}{723-T_{\mathrm{cold}}}\;$, $T_{\mathrm{avg}}=\frac{723+T_{\mathrm{cold}}}{2}$.

### SEM

The microstructures of the materials were analyzed using field‐emission scanning electron microscope (FE‐SEM S‐4800, Hitachi) at an accelerating voltage of 10 kV. Compositional analysis and backscattered electron (BSE) imaging were performed using a field‐emission SEM (SU‐8230, Hitachi) at an accelerating voltage of 10 kV equipped with a silicon‐drift EDX detector (Octane Elite Plus, EDAX). Ultrafine sample surfaces were prepared by Cooling Cross‐section Polisher (IB‐19520CCP, JEOL) using Ar^+^ ion. The acceleration voltage was 5 kV and the specimen stage was cooled using liquid nitrogen.

### STEM

The specimen for STEM analysis was prepared using a focused ion beam (FIB) system (Helios G5, FEI company) with a liquid gallium metal ion source. The specimen orientation was confirmed prior to the FIB process using electron backscatter diffraction equipped within a SEM (Quattro S, Thermo Fisher Scientific). The specimen orientation was maintained during the FIB process by preparing the sample in the plan‐view type. STEM was conducted using a spherical aberration‐corrected TEM (JEM‐ARM200CF, JEOL) at an acceleration voltage of 200 kV. The angle range of the HAADF detector was 68–280 mrad and the semi‐convergence angle was 24 mrad. The size of the electron probe was ≈0.8 Å. EDX elemental mapping was performed using dual‐type silicon‐drift EDX detectors (JED‐2300 T, JEOL Ltd.) with a large effective solid angle (Ω = ≈1.2 sr). The X‐ray sensing area of each detector was 100 mm^2^. Multiple frames of a 256 × 256 pixel chemical map up to more than 1500 frames were obtained with a probe size and beam current of ≈0.1 nm and ≈0.8 nA, respectively. The dwell time was 10 microsecond per pixel, and the acquisition time was 40 min. A Wiener filter in a weak mode was applied to reduce the statistical and background noise. The sample drift was corrected by tracking the reference position assigned at the beginning of the acquisition using the HAADF STEM image. An independent sample was also analyzed at the KAIST Analysis Center for Research Analysis (KARA) using spherical aberration‐corrected TEM (Titan Cubed G2 60–300, FEI Company) at an acceleration voltage of 300 kV with a spherical aberration corrector (CEOS GmbH) and a Gatan Image Filter (Quantum 965, Gatan Inc.). The size of the electron probe was ≈1 Å and the convergence semiangle was 17.9 mrad. EDX elemental mapping was performed with four integrated silicon‐drift EDX detectors. Electron energy‐loss spectra (EELS) were acquired for the low‐loss region to determine the sample thickness via the log‐ratio method. The average thickness of the region of interest was ≈40 nm, with a standard deviation of ±3 nm.

### XRD

Phase analysis was conducted using powder XRD with a high‐resolution X‐ray diffractometer (Smartlab, Rigaku) using a CuK*_*α*_* radiation source. The high‐temperature X‐ray diffraction pattern was acquired using a high‐temperature X‐ray diffractometer (D/MAX‐2500, Rigaku) under an N_2_ atmosphere at a heating rate of 5 K min^−1^. A platinum holder was used to prevent the reaction between the sample and holder.

### XPS

High‐temperature XPS was conducted using an in situ X‐ray photoelectron spectrometer (Axis‐Supra, Kratos) with a monochromatic Al X‐ray source. The binding energies of all the elements were calibrated to the adventitious carbon peak (284.8 eV). XPS fitting was performed using the least‐squares method to avoid any bias from manual fitting. Therefore, the reported spectra consist of a set of deconvoluted Lorentzian peaks that minimize the residual signals between the fitted curves and experimental data.

### Hall Measurements

Room‐temperature Hall measurements were conducted using a physical property measurement system (PPMS, Quantum Design) via the van der Pauw method under a magnetic field of 1T, 3T, and 5T. The high‐temperature Hall coefficients were measured using a custom‐built system under a magnetic field of 1.5 T using the van der Pauw method with a four‐probe setup. The temperature‐dependent carrier concentrations and mobilities were calculated using the Hall coefficient.

### Differential Scanning Calorimetry

Differential scanning calorimetry was conducted using a high‐temperature equipment (DSC 200 F3, Netzsch).

### Computational Details

First‐principles calculations were performed based on density functional theory with the generalized gradient approximation parameterized by Perdew, Burke, and Ernzerhof (PBE–GGA) implemented in the Vienna ab initio simulation package (VASP) code.^[^ ^39^ ^]^ We used the projector augmented wave (PAW) pseudopotential for valence and ionic core interactions.^[^ ^40^ ^]^ Cubic PbTe supercell structures with 64 atoms were used for the total energy calculations. An energy cutoff of 400 eV and a 4 × 4 × 4 gamma‐centered k‐point mesh were employed. The lattice parameter was fixed to 6.575 Å and the internal atomic positions were fully relaxed until the force was within 0.02 eV Å^−1^ for each atom. Spin orbital interactions were included in all calculations, except for the structural optimization.

## Conflict of Interest

The authors declare no conflict of interest.

## Supporting information

Supporting Information

## Data Availability

Research data are not shared.
