# Supplementary material for: Regulating Te Vacancies through Dopant Balancing via Excess Ag Enables Rebounding Power Factor and High Thermoelectric Performance in p‐Type PbTe
Source: Adv Sci (Weinh). 2021 Aug 13;8(20):2100895. doi: 10.1002/advs.202100895 (PMC8529492; doi:10.1002/advs.202100895)
Supplement: Supplementary file 1 — Supporting Information [file ADVS-8-2100895-s001.pdf]

WILEY-VCH

© 2021 Wiley-VCH GmbH

ADVANCED SCIENCE
Open Access

Supporting Information

for *Adv. Sci.,* DOI: 10.1002/advs.202100895

Regulating Te Vacancies through Dopant Balancing via Excess Ag Enables Rebounding Power Factor and High Thermoelectric Performance in p-Type PbTe

Hanhwi Jang, Jong Ho Park, Ho Seong Lee, Byungki Ryu, Su-Dong Park, Hyeon-Ah Ju, Sang-Hyeok Yang, Young-Min Kim, Woo Hyun Nam, Heng Wang, James Male, G. Jeffrey Snyder, Minjoon Kim, Yeon Sik Jung, and Min-Wook Oh**

Supporting Information

Regulating Te Vacancies through Dopant Balancing via Excess Ag Enables Rebounding Power Factor and High Thermoelectric Performance in p-Type PbTe

Hanhwi Jang, Jong Ho Park, Ho Seong Lee, Byungki Ryu, Su-Dong Park, Hyeon-Ah Ju, Sang-Hyeok Yang, Young-Min Kim, Woo Hyun Nam, Heng Wang, James Male, G. Jeffrey Snyder, Minjoon Kim, Yeon Sik Jung, and Min-Wook Oh**

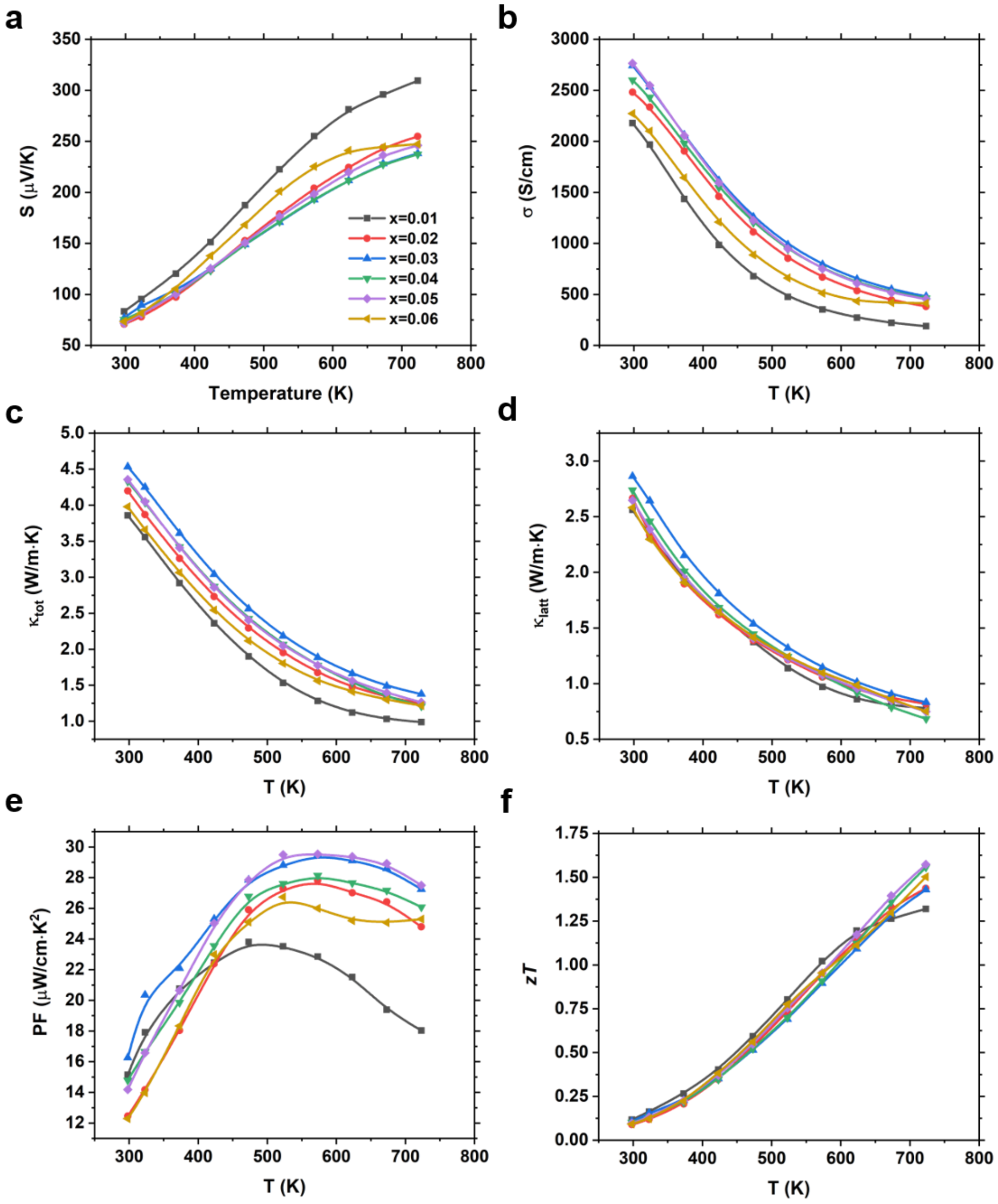

Figure S1. **Thermoelectric properties of $Na_xPb_{1-x}Te$.** Temperature-dependent (a) Seebeck coefficient (S). (b) electrical conductivity (σ), (c) total thermal conductivity (κ_{tot}), (d) lattice thermal conductivity (κ_{latt}), (e) power factor (PF), and (f) dimensionless figure of merit (*zT*). The same symbols indicate corresponding concentrations of Na.

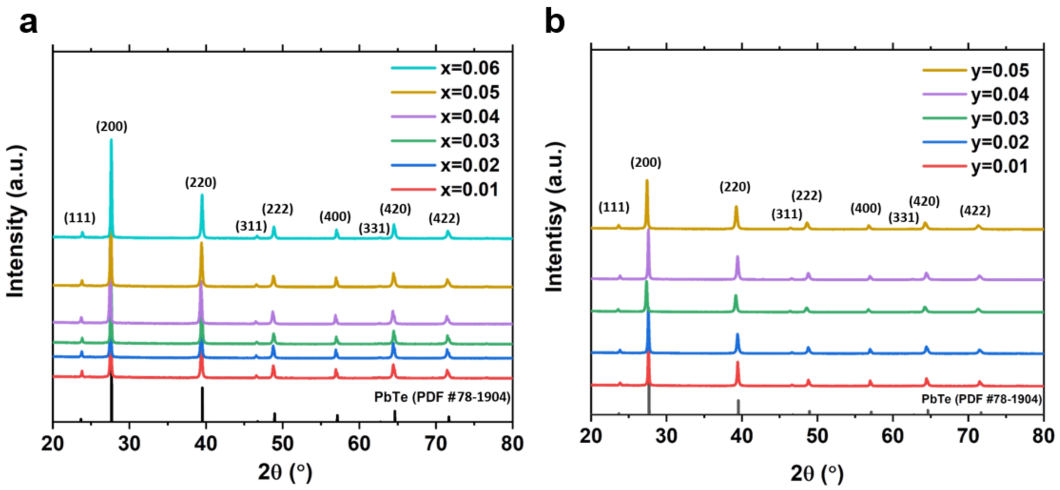

Figure S2. X-ray diffraction (XRD) pattern of (a) $Na_xPb_{1-x}Te$ and (b) $Na_{0.04}Pb_{0.96}Ag_yTe$ showing that secondary phases exist below a detection limit

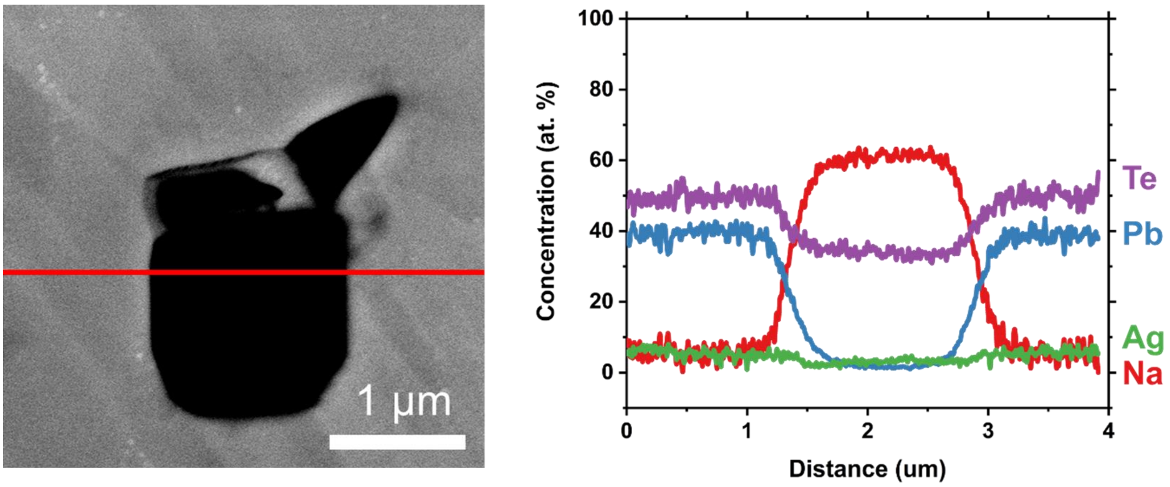

Figure S3. BSE image and corresponding SEM-EDX linescan profile of Na_2Te precipitates in $Na_{0.04}Pb_{0.96}Ag_{0.02}Te$.

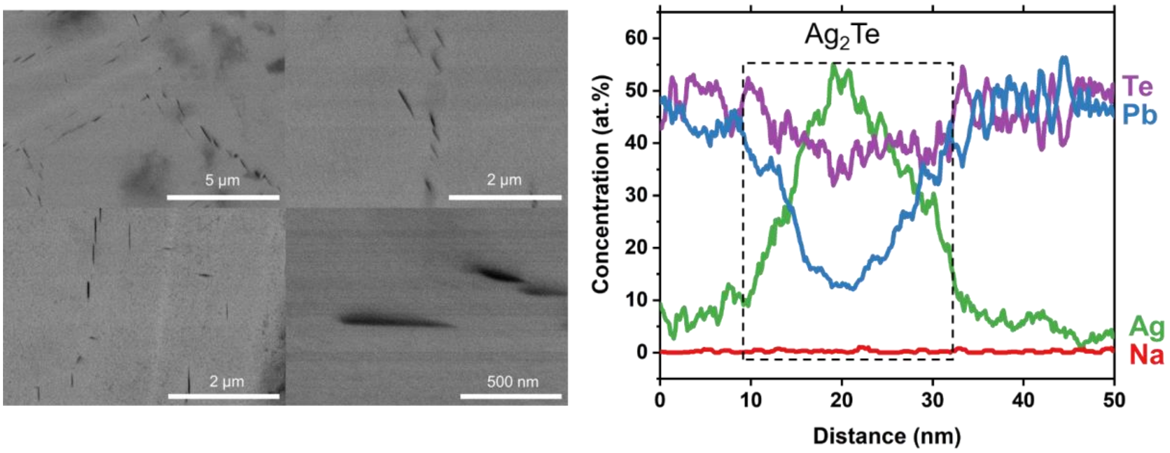

Figure S4. BSE images of the quaternary PbTe and STEM-EDX linescan profile. The dark short plates are Ag_2Te secondary phase having the length of few hundred nanometers and width of few tens of nanometers.

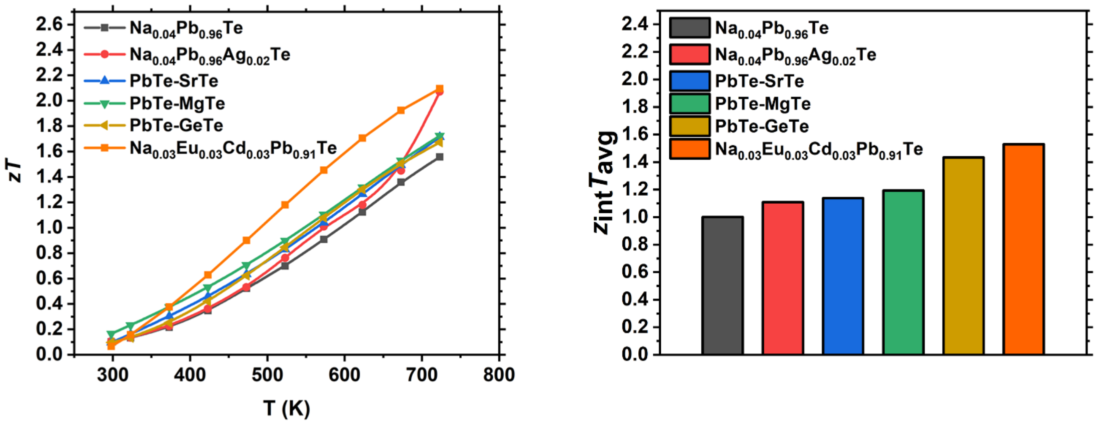

Figure S5. Comparison of temperature-dependent zT values and zT_{avg} at T_{cold} = 473 K with other high performance PbTe-based compounds[1-4, 10].

Table S1. Comparison of zT_{avg} values by various cold side temperatures. Hot side temperature is fixed to the 723 K

T_{cold} (K)	T_{avg}	$Z_{int}T_{avg}$					
		Na0.04Pb0.96Te	Na0.04Pb0.96Ag0.02Te	PbTe-SrTe	PbTe-MgTe	PbTe-GeTe	PbTe-EuTeCd
298	510.5	0.64	0.7	0.75	0.82	0.94	1.00
323	523	0.69	0.75	0.81	0.87	1.01	1.08
373	548	0.79	0.86	0.91	0.98	1.16	1.23
423	573	0.89	0.98	1.02	1.08	1.3	1.38
473	598	1.00	1.11	1.14	1.19	1.43	1.53
523	623	1.11	1.24	1.25	1.30	1.56	1.67

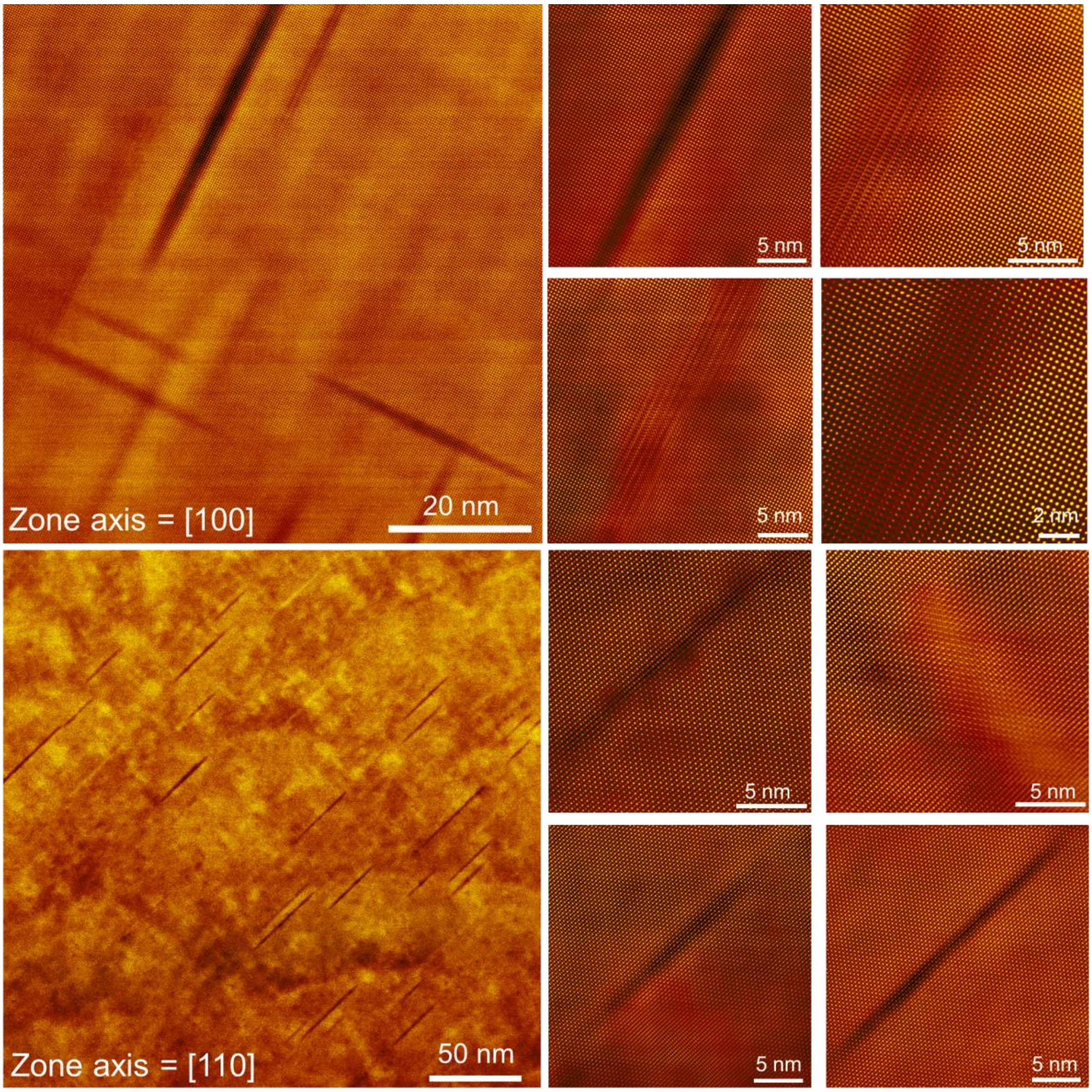

Figure S6. HAADF STEM images of $Na_{0.04}Pb_{0.96}Ag_{0.02}Te$ viewed along [100] and [110] zone axis. This sample was analyzed in KAIST Analysis center for Research Analysis (KARA) by FEI Titan Cubed G2 Cs-corrected STEM under an acceleration voltage of 300 kV. The morphology and contrast of needle-like precipitates and Ag interstitials were consistent to the results obtained using JEOL JEM-ARM200CF.

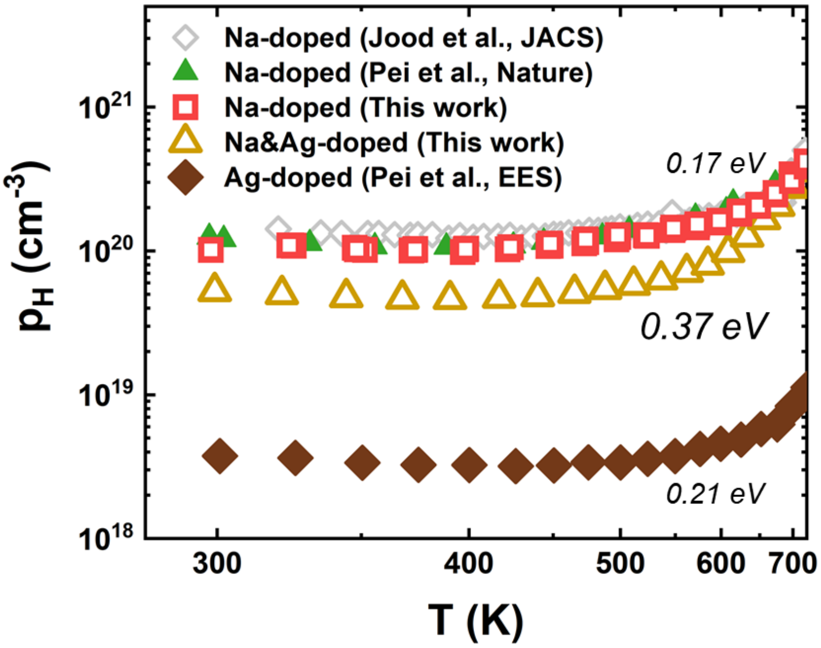

Figure S7. Comparison of high-temperature Hall carrier concentration for PbTe-based self-tuning system and its activation energy at high temperatures.

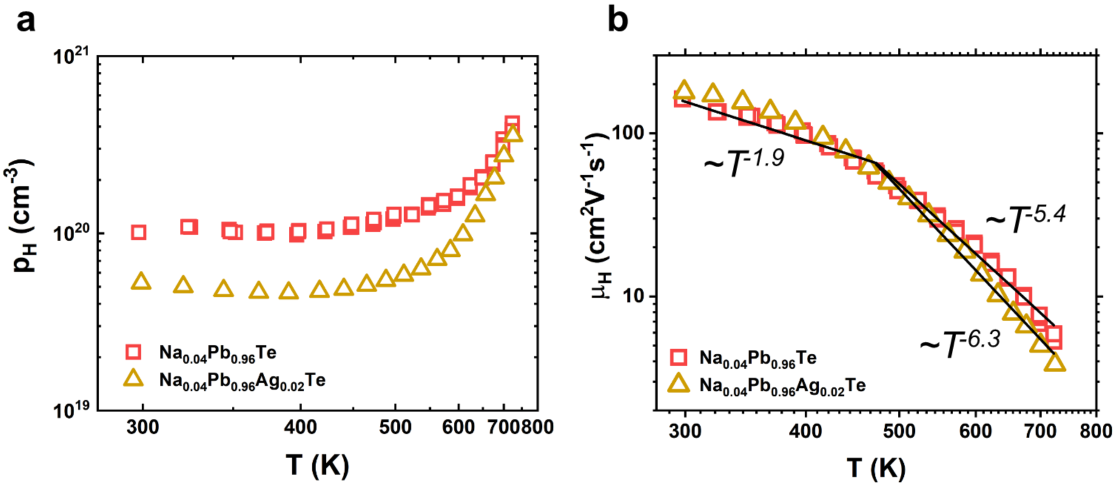

Figure S8. High-temperature (a) Hall carrier concentration and (b) Hall mobility of $Na_{0.04}Pb_{0.96}Ag_{0.02}Te$ and $Na_{0.04}Pb_{0.96}Te$. Strong temperature dependence of the mobility results from the participation of heavy holes in the conduction process.

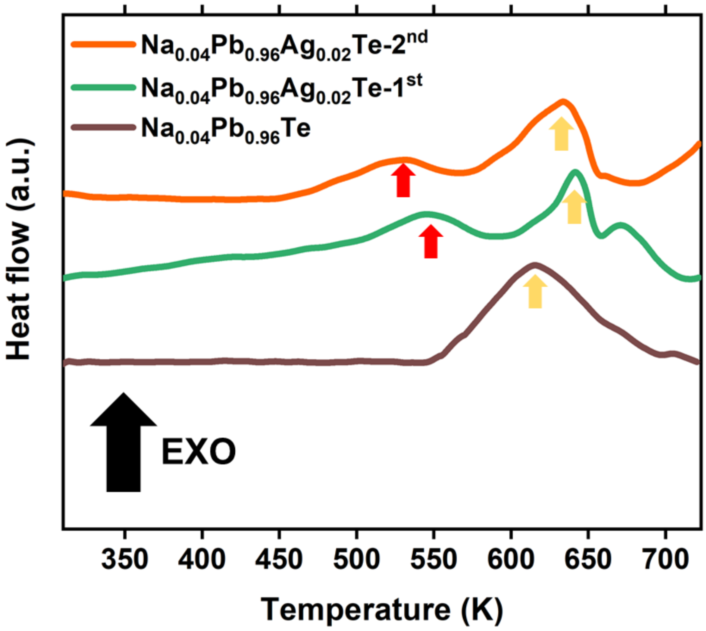

Figure S9. Differential scanning calorimetry (DSC) results showing two exothermic peaks observed in quaternary $Na_{0.04}Pb_{0.96}Ag_{0.02}Te$ during two heating cycles. However, only one peak near 623 K is observed in $Na_{0.04}Pb_{0.96}Te$ corresponding to the Na re-dissolution. Another peak near 550 K is owing to the redistribution of Ag.

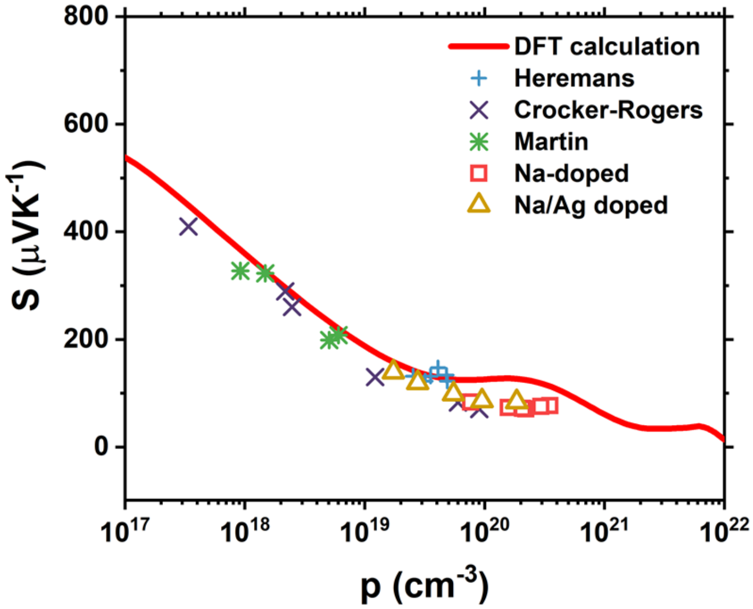

Figure S10. Carrier concentration-dependent Seebeck coefficient (Pisarenko plot) of PbTe-based thermoelectric materials.

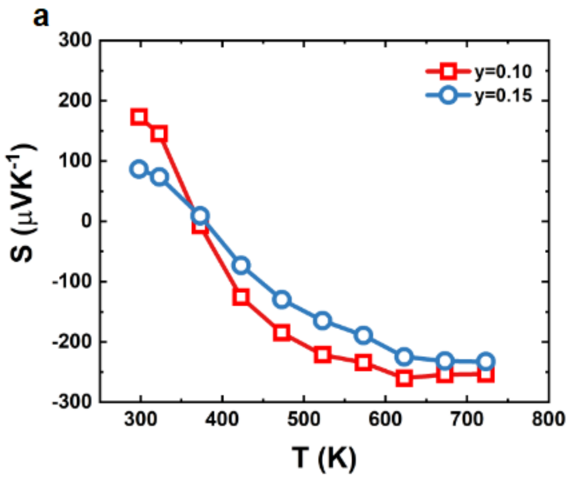

Figure S11. Electronic transport properties of $Na_{0.04}Pb_{0.96}Ag_yTe$ (y=0.1 and 0.15) Temperature-dependent (a) Seebeck coefficient and (b) electrical conductivity showing a p- to n-type transition at 390 K. Non-degenerate semiconducting characteristics are observed after the transition in electrical conductivity.

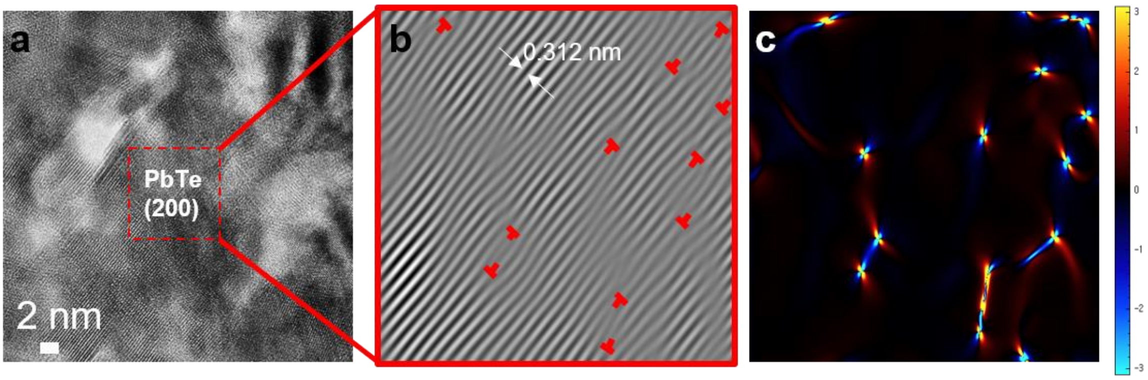

Figure S12. TEM characterization of $Na_{0.04}Pb_{0.96}Ag_{0.02}Te$ (a) High-resolution transmission electron microscopy (HRTEM) image of $Na_{0.04}Pb_{0.96}Ag_{0.02}Te$. (b) Fast Fourier transformation (FFT) image of square shown in (a), revealing that high density of dislocations exist in the PbTe lattice. (c) Geometric phase analysis showing lattice strain in xx-direction (ε_{xx}).

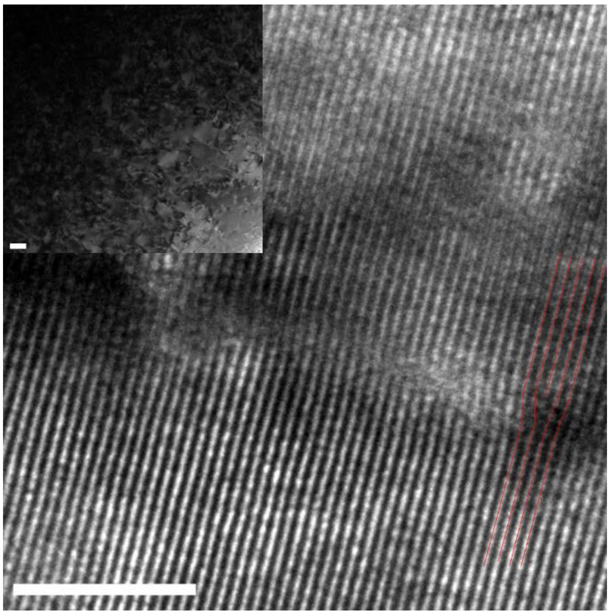

Figure S13. HRTEM image of $Na_{0.04}Pb_{0.96}Ag_{0.02}Te$ showing edge dislocations in the lattice. Lattice planes and an extra half plane were shown by red solid lines. Scale bar = 5 nm. Inset is the low-magnification TEM image of the same sample showing a number of dislocation loops. Scale bar = 100 nm.

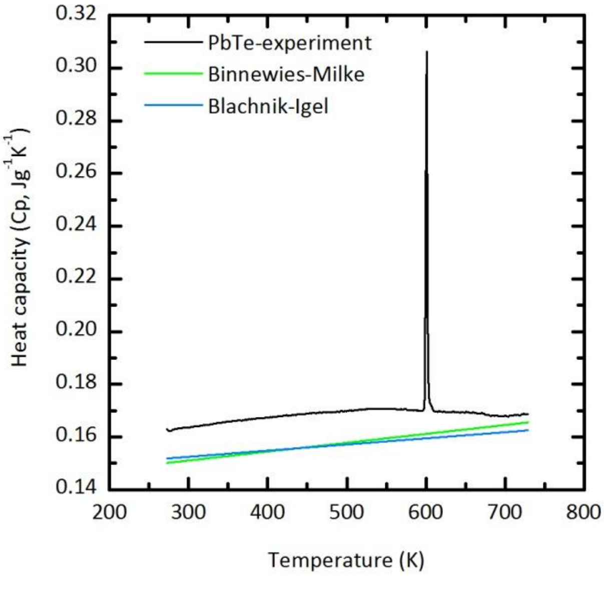

Figure S14. Measured heat capacity of the binary PbTe. A comparison of the measured heat capacity of the binary PbTe with the typical fitting values of lead chalcogenides. A constant

heat capacity value of 0.171 J/gK was used for the calculation of the total thermal conductivity. The endothermic peak in the binary arose from Pb residues.

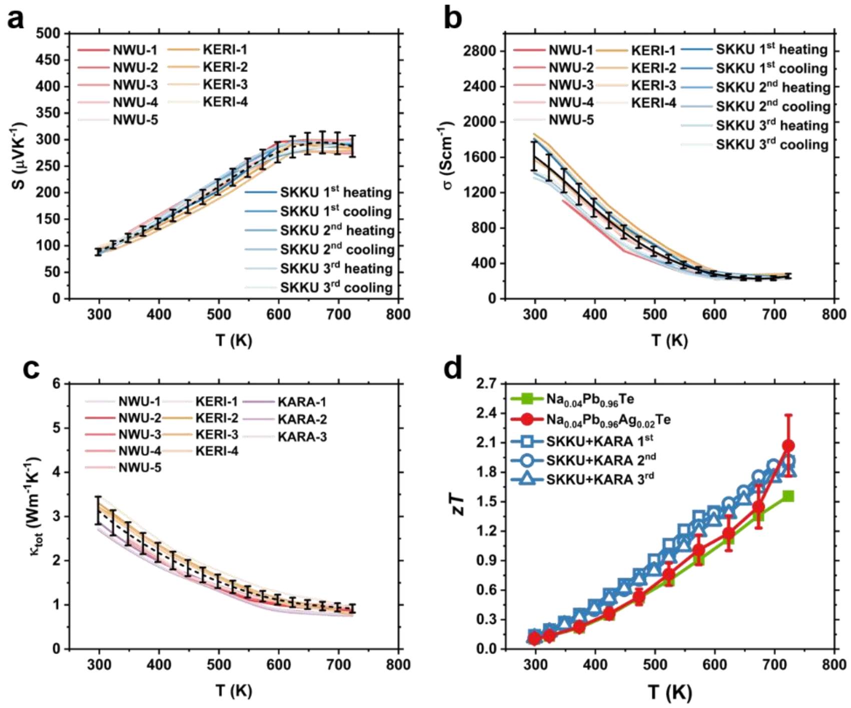

Figure S15. The temperature-dependent (a) Seebeck coefficient, (b) electrical conductivity, (c) total thermal conductivity, and (d) *zT* of $Na_{0.04}Pb_{0.96}Ag_{0.02}Te$ measured from multiple institutes. The thermoelectric property of the sample is cross-checked at KERI, Northwestern University (NWU), Sungkyunkwan University (SKKU) and KAIST Analysis center for Research Advancement (KARA) with multiple measurements. Black dashed lines indicate an average of measured values.

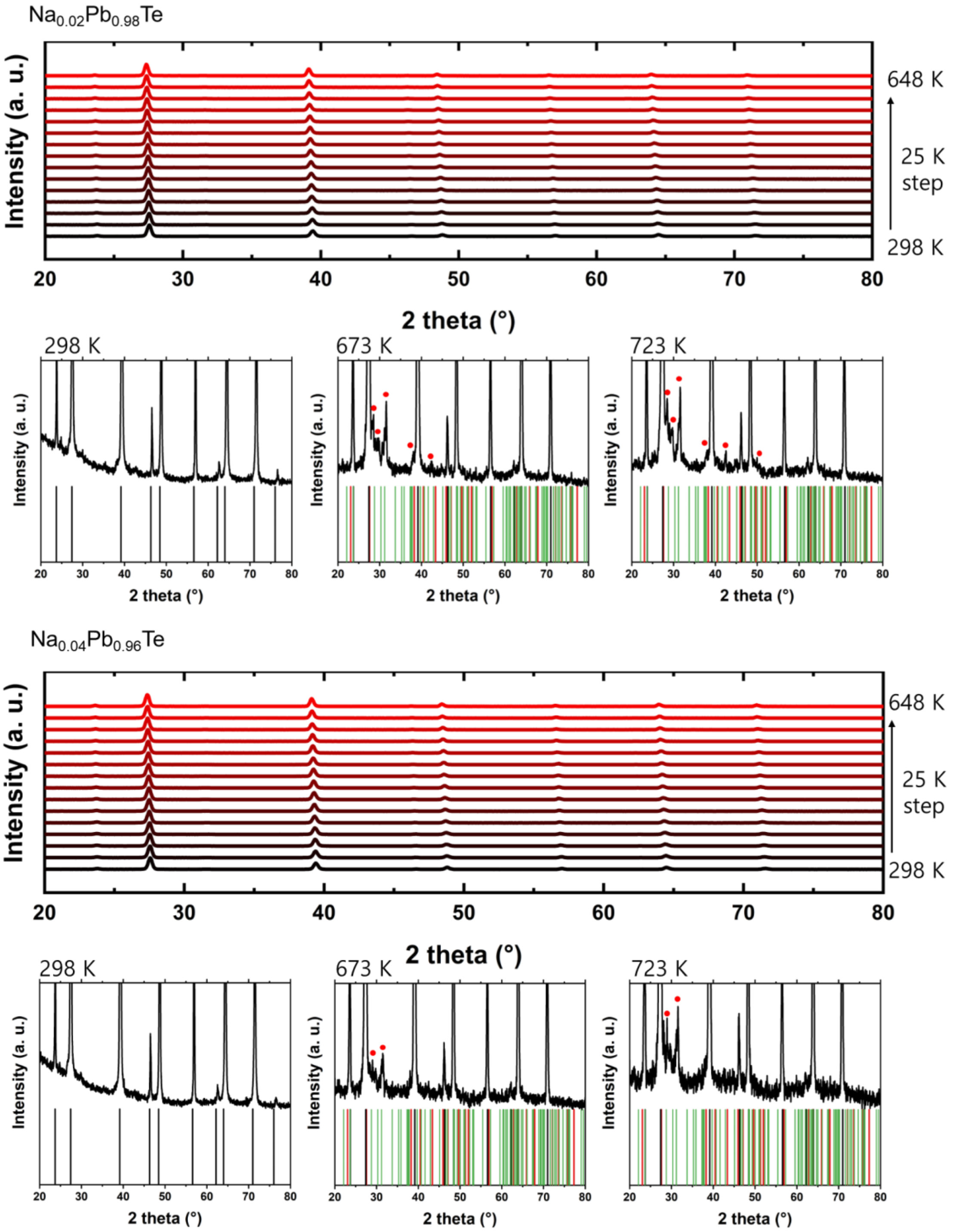

Figure S16. High-temperature powder XRD patterns of $Na_{0.02}Pb_{0.98}Te$ and $Na_{0.04}Pb_{0.96}Te$. Metallic Te (PDF # 01-071-3934; monoclinic, green; PDF # 01-078-2312; trigonal, red) precipitates were observed above 673 K. Other peaks were assigned to the cubic PbTe (PDF #01-080-5251, black). Red circles denote the peaks from metallic Te.

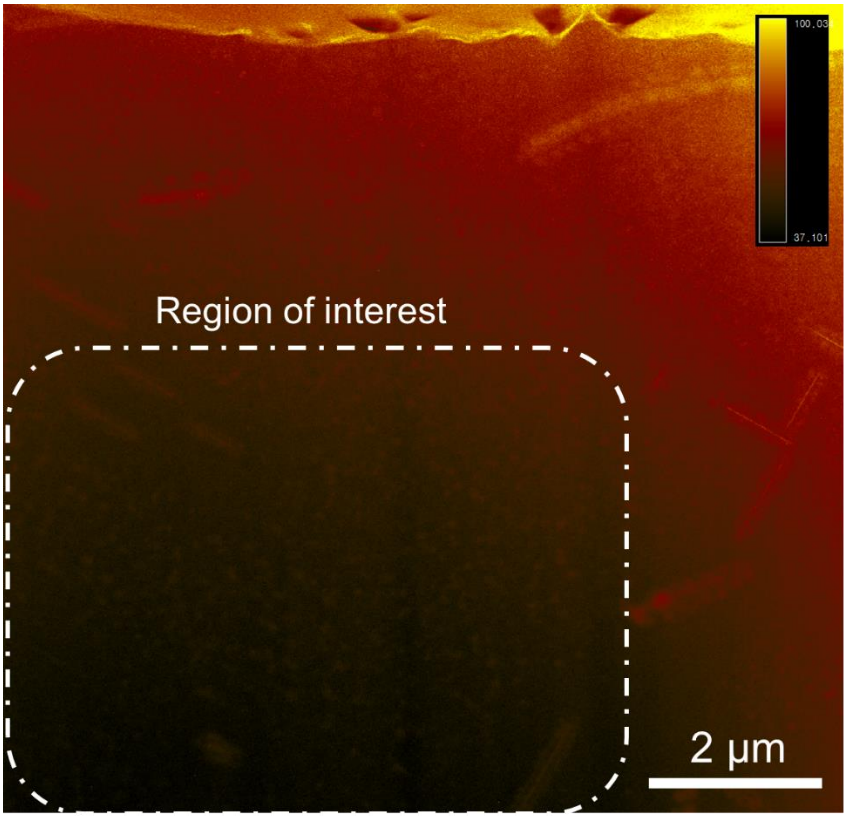

Figure S17. Thickness map of the TEM specimen measured by EELS. Upper region of sample was thicker than lower region to prevent any structural deformation during milling or TEM observation.

Calculation of electronic transport properties

A theoretical electronic transport property is obtained by a systematic calculation of Boltzmann transport equation. We used three band model with non-parabolic Kane band to consider the effect of band convergence, bipolar conduction, and the non-parabolicity of the valence band in PbTe to the transport properties. Thus, the dispersion relation $\mathrm{E}\left(1+\frac{E}{E_g}\right)=\frac{\hbar^2k^2}{2m_d^*}$ is used in the calculation where E is energy, E_g is band gap, $\hbar$ is reduced Planck constant, k is wave vector, and $m_d^* = (m_l^* m_t^{*2})^{1/3}$ is the density-of-state effective mass of the each valley. To reflect the simultaneous contribution of light, heavy holes and electrons on the transport properties, the effective mass of three bands are set differently.

The major scattering mechanism at the temperature range of this study is deformation potential scattering, where a relaxation time is given by following equation:

$$\tau_{ac}(\varepsilon) = (\epsilon+\alpha\varepsilon^2)^r(1+2\alpha\varepsilon)^{-1}\frac{2\pi\hbar^4C_l}{\Xi_{ac}^2(2m_d^*k_BT)^{3/2}[(1-A)^2-B]},$$

where $\varepsilon = E/k_BT$ is reduced carrier energy, $\alpha = k_BT/E_g$ is pre-factor for non-parabolicity of the band, r=-1/2 for acoustic phonon scattering, and $A = \alpha\varepsilon(1-K)/(1+2\alpha\varepsilon)$, $\mathrm{B} = 8\alpha\varepsilon(1+\alpha\varepsilon)K/[3(1+2\alpha\varepsilon)^2]$, $K = (E_{ac})_v/(E_{ac})_c \approx 1$. Furthermore, the effect of Na and Ag doping is considered by perturbating the total relaxation time by point defect scattering. The point defect scattering relaxation time is given by:

$$\tau_{PD}(\varepsilon) = (\varepsilon+\alpha\varepsilon^2)^r(1+2\alpha\varepsilon)^{-1}\frac{\pi\hbar^4}{\Delta^2(2m_d^*k_BT)^{3/2}N_d[(1-A)^2-B]},$$

where r is equal to -1/2 for acoustic scattering, N_d is the density of point defects. The total relaxation time is calculated according to the Matthiessen's rule as: $\tau^{-1}(\varepsilon) = \tau_{ac}^{-1}(\varepsilon) + \tau_{PD}^{-1}(\varepsilon)$.

The electrical conductivity and Seebeck coefficient are calculated by assuming energy dependent relaxation time approximation as given by following equation:

$$\sigma = \frac{e^2}{m_I^*}\frac{(2m_d^* k_B T)^{3/2}}{3\pi^2\hbar^3}\langle\tau(\varepsilon)\rangle,$$

$$S = \frac{k_B}{e}\frac{\langle\tau(\varepsilon)(\varepsilon - \varepsilon_F)\rangle}{\langle\tau(\varepsilon)\rangle},$$

where k_B is Boltzmann constant, e is the elementary charge of the electron, $m_I^* = 3(1/m_l^* + 2/m_t^*)^{-1}$ is the inertial effective mass, and $\varepsilon_F = E_F/k_B T$ is the reduced Fermi energy. $\langle\tau(\varepsilon)\rangle$ is defined as an average of the relaxation time $\tau(\varepsilon)$ over energy ε, which is given by:

$$\langle\tau(\varepsilon)\rangle = \int_0^\infty (\varepsilon + \alpha\varepsilon^2)^{3/2}(1 + 2\alpha\varepsilon)^{-1}\left(\frac{\partial f_0}{\partial\varepsilon}\right)\tau(\varepsilon)d\varepsilon,$$

Where f_0 is the Fermi-Dirac distribution function at equilibrium.

We assumed the mass anisotropy of each band $\mathrm{b} = \frac{m_l^*}{m_t^*} \approx 1$ in the calculation so that the inertial effective mass is equal to the density-of-state effective mass, which describes the experimental data with reasonable accuracy. The total electrical conductivity is calculated by considering the contributions from each band given by:

$$\sigma = \sigma_L + \sigma_\Sigma + \sigma_C$$

$$S = \frac{S_L\sigma_L + S_\Sigma\sigma_\Sigma + S_C\sigma_C}{\sigma}$$

Table S2. Parameters used to calculate the electronic transport properties

Symbols	Description	Value[5,6]
m_C^*	C band effective mass	0.3 m_0
m_L^*	L band effective mass	0.36 m_0

m_{Σ}^{*}	Σ band effective mass	2.1 m_0
$\Delta E_{C\text{-}L}$	Band offset between C band and L band	0.18+0.0004T (eV)
$\Delta E_{C\text{-}\Sigma}$	Band offset between C band and Σ band	0.42 (eV)
$\Xi_{ac,C,L}$	Deformation potential of C band and L band	22 eV
$\Xi_{ac,\Sigma}$	Deformation potential of Σ band	17 eV
C_l	Elastic modulus of C, L, and Σ band	77 GPa
Δ	Point defect scattering potential	2.5 eV

Calculation of lattice thermal conductivity

The theoretical lattice thermal conductivity is calculated based on the modified Debye-Callaway model:

$$\kappa_{latt} = \frac{k_B}{2\pi^2 \upsilon}\left(\frac{k_B T}{\hbar}\right)^3 \int_0^{\frac{\theta_D}{T}} \tau(z)\, \frac{z^4 \exp(z)}{[\exp(z)-1]^2} dz,$$

where υ is the average sound velocity and θ_D is Debye temperature of PbTe. For determination of relaxation time, Umklapp process, normal process, scattering by dislocation core, dislocation strain, and point defects are considered in the calculation. From the observation of the grain size (about 80 μm), it is expected that the effect of grain boundary scattering on the lattice thermal conductivity is negligible. Therefore, we do not consider the boundary scattering mechanism. The relaxation time of each process is given as follows:

$$\tau_U^{-1} + \tau_N^{-1} = B\omega^2 T exp\left(-\frac{\theta_D}{3T}\right) \text{ (Umklapp and normal process)}$$

$$\tau_{DC}^{-1} = N_D \frac{\bar{V}^{4/3}}{\upsilon^2}\omega^3 \text{ (Dislocation core)}$$

$$\tau_{DS}^{-1} = CB_D^2 N_D \gamma^2 \omega \left[\frac{1}{2} + \frac{1}{24}\left(\frac{1-2r}{1-r}\right)^2 \left(1 + \sqrt{2}\left(\frac{\upsilon_L}{\upsilon_T}\right)^2\right)^2\right] \text{ (Dislocation strain)}$$

$$\tau_{PD}^{-1} = \frac{\bar{V}\omega^4}{4\pi\upsilon^3}\Gamma \text{ (Point defects)}$$

The total relaxation time is given by Matthiessen's rule as: $\tau^{-1} = \tau_U^{-1} + \tau_N^{-1} + \tau_{DC}^{-1} + \tau_{DS}^{-1} + \tau_{PD}^{-1}$.

The pre-exponential factor for the Umklapp process, *B*, was determined from the ternary PbTe. For the quaternary system, the pre-factor for dislocation strain C is set to 5 as reported in the literature[7], which attributes such a high C value to the strengthening of mass fluctuation due to the segregation of Ag near dislocation in Ag-doped PbTe system. As the dopant element and concentration is similar, the calculation fits excellently to the experimental data. The scattering parameter for point defect (Γ) is calculated according to the previously reported method[8], which explains the abrupt drop of lattice thermal conductivity above 623 K in terms of increased point defect density.

Table S3. Parameters used to calculate the lattice thermal conductivity

**Symbols**	**Description**	**Value[7,8]**
υ	Average sound velocity	1770 m/s
N_D	Dislocation density of $Pb_{0.96}Na_{0.04}Ag_{0.02}Te$	$2\times10^{10}/cm^2$
B_D	Magnitude of Burgers vector	4.57×10^{-10} m
C	Dislocation scattering pre-factor	5
γ	Grüneisen parameter	1.96
υ_L	Longitudinal sound velocity	3590 m/s
υ_T	Transverse sound velocity	1610 m/s
r	Poisson's ratio	0.218
$\boldsymbol{\theta_D}$	Debye temperature	136 K

Supplementary Note 1. Defect chemistry in ternary and quaternary PbTe

It is known that the ternary PbTe with excess Na contains Na_2Te-related secondary phase in the matrix due to low solid solubility of Na at room temperature. For example, 4% Na-doped PbTe will form Na_2Te phase as follows:

$$(RT)\ \mathrm{Na}_{0.04}\mathrm{Pb}_{0.96}Te \rightarrow x\cdot Na_2Te + Na_{0.04-2x}Pb_{0.96}Te_{1-x}\ (x \leq 0.02)$$

where V_{Pb}, Na_{Pb}, and V_{Te} are the predominant defects in the system. The resulting charge compensation occurs by following equation:

$$2x\cdot V_{Pb}^{2-} + 2x\cdot 2\cdot h^{+} + (0.04-2x)\cdot Na_{Pb}^{-} + (0.04-2x)\cdot h^{+} + x\cdot V_{Te}^{2+} + x\cdot 2\cdot e^{-} = 0.04\cdot h^{+}$$

However, the solid solubility of Na increases at high temperatures, providing Na to the matrix by following equation:

$$(HT)\ x\cdot Na_2Te + Na_{0.04-2x}Pb_{0.96}Te_{1-x} \rightarrow Na_{0.04}Pb_{0.96}Te_{1-x} + x\cdot Te^{0}$$

Here, Te^{0} stands for metallic Te that is not incorporated into the PbTe matrix. This postulation is consistent with experimental observation of Jood et al. where metallic Te precipitates emerge at high temperature.[9] The charge compensation at high temperatures is as follows:

$$0.04\cdot Na_{Pb}^{-} + 0.04\cdot h^{+} + x\cdot V_{Te}^{2+} + x\cdot 2\cdot e^{-} = (0.04-2x)\cdot h^{+}$$

Hence, remaining Te would reduce hole concentration compared to room temperature.

On the other hand, the quaternary PbTe ($Na_{0.04}Pb_{0.96}Ag_{0.02}Te$) also contains Ag_2Te as well as Na_2Te. Its corresponding mass balance equation is as follows:

$$(RT)\ \mathrm{Na_{0.04}Pb_{0.96}Ag_{0.02}}Te \rightarrow x \cdot Na_2Te + y \cdot Ag_2Te + Na_{0.04-2x}Pb_{0.96}Ag_{0.02-2y}Te_{1-x-y}\ (x \leq 0.02, y \leq 0.01)$$

where V_{Pb}, Na_{Pb}, V_{Te}, and Ag_i is the predominant defect. Similar to the ternary system, its charge compensation equation is as follows:

$$2x \cdot V_{Pb}^{2-} + 2x \cdot 2 \cdot h^+ + (0.02 - 2y) \cdot Ag_i + (0.02 - 2y) \cdot e^- + (0.04 - 2x) \cdot Na_{Pb}^- + (0.04 - 2x) \cdot h^+ + (x + y) \cdot V_{Te}^{2+} + (x + y) \cdot 2 \cdot e^- = 0.02 \cdot h^+$$

Thus, the excess doping of Ag will reduce the hole concentration which is in good agreement with our experiment.

At high temperatures, as supported by our experimental measurements, Na enters to the Pb sites and Ag enters to the interstitial sites. Compared to the ternary system, the quaternary system does not show any trace of metallic Te from in situ XRD measurement. Considering the incorporation of Te yields the following mass and charge balance equation.

$$(HT)\ x \cdot Na_2Te + y \cdot Ag_2Te + Na_{0.04-2x}Pb_{0.96}Ag_{0.02-2y}Te_{1-x-y} \rightarrow Na_{0.04}Pb_{0.96}Ag_{0.02}Te$$

$$0.04 \cdot Na_{Pb}^- + 0.04 \cdot h^+ + 0.02 \cdot Ag_i + 0.02 \cdot e^- = 0.02 \cdot h^+$$

From these calculations, one can notice that the presence of metallic Te and V_{Te} is limiting the hole concentration, whereas the presence of both is suppressed in the quaternary. The detailed mechanism of the enhanced solubility of Te in the quaternary should be studied further.

References

1 Zhao, L. D. *et al.* All-scale hierarchical thermoelectrics: MgTe in PbTe facilitates valence band convergence and suppresses bipolar thermal transport for high

performance. *Energy & Environmental Science* **6**, 3346-3355, doi:10.1039/c3ee42187b (2013).

2 Biswas, K. *et al.* High-performance bulk thermoelectrics with all-scale hierarchical architectures. *Nature* **489**, 414-418, doi:10.1038/nature11439 (2012).

3 Chen, Z. *et al.* Lattice Dislocations Enhancing Thermoelectric PbTe in Addition to Band Convergence. *Adv Mater* **29**, 1606768, doi:10.1002/adma.201606768 (2017).

4 Jood, P., Ohta, M., Yamamoto, A. & Kanatzidis, M. G. Excessively Doped PbTe with Ge-Induced Nanostructures Enables High-Efficiency Thermoelectric Modules. *Joule* **2**, 1339-1355, doi:10.1016/j.joule.2018.04.025 (2018).

5 Wu, D. *et al.* Superior thermoelectric performance in PbTe-PbS pseudo-binary: extremely low thermal conductivity and modulated carrier concentration. *Energy & Environmental Science* **8**, 2056-2068, doi:10.1039/c5ee01147g (2015).

6 Zhang, Q. *et al.* Heavy doping and band engineering by potassium to improve the thermoelectric figure of merit in p-type PbTe, PbSe, and PbTe(1-y)Se(y). *J Am Chem Soc* **134**, 10031-10038, doi:10.1021/ja301245b (2012).

7 Yu, Y. *et al.* Ag-Segregation to Dislocations in PbTe-Based Thermoelectric Materials. *ACS Appl Mater Interfaces* **10**, 3609-3615, doi:10.1021/acsami.7b17142 (2018).

8 Koh, Y. K., Vineis, C. J., Calawa, S. D., Walsh, M. P. & Cahill, D. G. Lattice thermal conductivity of nanostructured thermoelectric materials based on PbTe. *Applied Physics Letters* **94**, 153101, doi:10.1063/1.3117228 (2009).

9 Jood, P. *et al.* Na Doping in PbTe: Solubility, Band Convergence, Phase Boundary Mapping, and Thermoelectric Properties. *Journal of the American Chemical Society* **142**, 15464-15475, doi:10.1021/jacs.0c07067 (2020).

10 Wu, Y. *et al.* Thermoelectric Enhancements in PbTe Alloys Due to Dislocation-Induced Strains and Converged Bands. *Advanced Science* **7**, 1902628, doi:10.1002/advs.201902628 (2020).
